# Reliability and validity testing of the Canadian agility and movement skill assessment test and establishment of the shanghai norm

**DOI:** 10.3389/fpsyg.2026.1833662

**Published:** 2026-08-20

**Authors:** Haitan Wu, Mengfei Zhang

**Affiliations:** School of Physical Education Shanghai Normal University, Shanghai, China

**Keywords:** Canadian agility and movement skill assessment, children, reliability, Shanghai Norm, validity

## Abstract

**Background:**

Childhood is crucial for students’ FMS development, and the growth of these skills is also highly important for their adulthood development. Offering an effective, dependable assessment tool can not only accurately measure students’ FMS development but also offer specific guidance to help them enhance their skills.

**Methods:**

CAMSA was conducted with a sample of 1,183 students from five districts in Shanghai, with an average age of 9.98 ± 1.42 years. This study adopted specific methods and procedures for assessing the reliability and validity of measurement tools. Two indicators were selected for reliability: test-retest reliability and rater reliability. For validity, three indicators were chosen: structural validity, content validity, and concurrent validity—in addition to the subsequent construction of age-specific percentile norms for Shanghai children via standardized Z-score transformation.

**Results:**

The revised CAMSA demonstrated a reliability coefficient between 0.704 and 0.874, with the time score at 0.777, skill score at 0.814, and total score at 0.850. Rater reliability ranged from 0.678 to 0.922. The difficulty scores ranged from 0.52 to 0.71, with skill score difficulty between 0.64 and 0.77, and time score difficulty from 0.39 to 0.64. The skill and time scores were 0.71 and 0.52, respectively, indicating appropriate difficulty levels. The differentiation values for the total CAMSA score, skill score, and time score were 0.35, 0.34, and 0.52, respectively, suggesting effective differentiation. The scale also demonstrated acceptable structural validity, with fit indices meeting the pre-specified thresholds (χ^2^/df = 3.849, CFI = 0.931, GFI = 0.988, AGFI = 0.975, NFI = 0.911, RMR = 0.012, RMSEA = 0.050). Furthermore, the mean CAMSA score was 17.82 with a standard deviation of ± 3.80, while the average TGMD-3 score was 56.57 with a standard deviation of ± 22.97. The correlation coefficient between the TGMD-3 and CAMSA scores was 0.613(*P* = 0.000), the corresponding coefficient of determination R^2^ was reported as 0.376.

**Conclusion:**

The CAMSA is a reliable and valid assessment tool for evaluating fundamental movement skills in children aged 8–12 years. The study established norms from grades 1 to 5 and percentile norms that can be used to assess the development of children’s fundamental movement skills.

## Highlights

1. This study provides a foundation for Shanghai to develop standards for evaluating students’ agility and movement skills.
2. It fills a geographical gap in global adolescent agility and movement skills, contributing new data from an underrepresented region of China.
3. The findings support future public-health planning and policy development for the healthy development of children in Shanghai and similar settings.

## 1 Introduction

The acquisition and refinement of fundamental movement skills (FMS) during childhood constitute a critical public health priority, as they establish the foundational motor competencies necessary for lifelong physical activity engagement and holistic health development (Larsen et al., 2015; Robinson et al., 2015). Empirical evidence consistently demonstrates that proficient FMS in childhood is positively associated with increased participation in both organized and free-play sports activities, effectively attenuates the high sedentary time often concomitant with academic pressures, and confers sustainable benefits across multiple health domains (Barnett et al., 2010; Robinson et al., 2015). These benefits include the facilitation of habitual physical activity, maintenance of healthy body composition, and enhancement of cardiovascular fitness—collectively contributing to improved health trajectories across the lifespan (Barnett et al., 2008; Roberts et al., 2012).

Childhood, particularly the primary school years, is universally acknowledged as the sensitive period for FMS development. The level of FMS proficiency attained during this phase not only predicts immediate motor performance but also serves as a significant correlate of physical activity levels, motor competence, and associated health outcomes in adolescence and adulthood. Therefore, the availability of a valid, reliable, and feasible assessment tool is imperative. Such an instrument enables the objective evaluation of children’s FMS status, informs the development of evidence-based physical education curricula and targeted intervention programs, and facilitates population-level monitoring of motor development.

In the Chinese context, the recent integration of FMS benchmarks into the national Compulsory Education Physical Education and Health Curriculum Standards (2022 Edition) underscores the growing institutional recognition of FMS as a core component of child development (Ministry of Education, 2022). This policy shift necessitates the adoption of robust assessment methodologies (Barnett et al., 2016). While several FMS assessment tools (e.g., TGMD, KTK, MABC, AST) (Diao et al., 2018; Hua et al., 2010; Li and Ma, 2007; Li et al., 2022; Li et al., 2023; Ning et al., 2021)have been adapted for use in China, most evaluate skills in isolation, lacking the ecological validity of assessing skills in combination under time constraints, as required in many sports and games (Chang et al., 2020).

The Canadian Agility and Movement Skill Assessment (CAMSA) was developed to address this gap. As a circuit-based, performance-oriented assessment, the CAMSA uniquely evaluates the integration of fundamental movement skills with agility in a dynamic, ecologically valid context that mirrors real-world sport and play (Hoeboer et al., 2018; Longmuir et al., 2017). Although its psychometric properties have been established in several Western countries (Dania et al., 2020; Kaioglou et al., 2020; Lander et al., 2017; Menescardi et al., 2022), a comprehensive validation and norming study within a large, representative sample of Chinese children is lacking (Cao, 2020; Chang et al., 2021; Li et al., 2022; Mao et al., 2026; Mao et al., 2026). Specifically, while existing local studies have offered preliminary support for partial core psychometric features of the CAMSA, critical evidence including robust structural validity data, systematic cross-subgroup measurement invariance testing, and the development of population-specific percentile norms for the Chinese pediatric population still remains insufficient and far from comprehensive.

Therefore, the present study aimed to: (1) conduct a comprehensive psychometric validation (including structural validity, and reliability) of the CAMSA in a large sample of school-aged children in Shanghai, China; and (2) establish the first set of sex- and age-specific percentile norms for the CAMSA based on this population. By addressing these dual objectives, this study seeks to provide researchers and practitioners in China with a rigorously validated, contextually relevant tool for assessing integrated movement competence, thereby supporting the national goal of fostering physically literate and active youth.

## 2 Materials and methods

### 2.1 Test objects

The study participants were 8–12-year-old primary school students (mean age = 9.98 ± 1.42 years) recruited from five geographically diverse schools across Shanghai. A stratified random sampling design was adopted, with all eligible schools officially registered by the local education bureau as the complete sampling frame. Pre-randomized school sequences were generated within each stratum, and non-participating schools were sequentially replaced by pre-selected backups to maintain equal a priori selection probability. The five schools were selected through stratified random sampling, with 2 schools randomly drawn from urban areas, 2 from suburban areas, and 1 from the urban-suburban fringe of Shanghai. All participants were healthy, had no contraindications to exercise, and could complete the test. By the principle of voluntary participation, students who could not continue for any reason were allowed to withdraw. A total of 1,183 students participated in the test; however, 23 students were excluded due to missing data from their absence. Consequently, the final valid sample comprised 1160 participants, including 587 boys and 573 girls. Disaggregated by single-year age strata, the sample distribution was as follows: 113 boys and 116 girls in the 8-year-old group, 131 boys and 124 girls in the 9-year-old group, 109 boys and 113 girls in the 10-year-old group, 109 boys and 110 girls in the 11-year-old group, and 125 boys and 110 girls in the 12-year-old group.

### 2.2 Testing tools

#### 2.2.1 CAMSA test

According to the standard procedures outlined in the CAMSA test manual, the test will be conducted from September to December 2023. All testers involved in this study possess extensive experience in movement skill analysis. The team consists of five graduate students majoring in sports from the School of Physical Education at Shanghai Normal University. Each tester has completed a total of 10 h of training to fulfill the test requirements. To ensure the safety of the participants, subjects will engage in a 5-min warm-up activity before each test. The testing will take place either on the school playground or in the indoor sports hall. The CAMSA is a circuit-based test for children aged 8–12 years consists of seven movement items: 2-foot jumping (2 points), sliding (3 points), catching (1 point), throwing (2 points), skipping (2 points), one-foot hopping (2 points), and kicking (2 points).

The research and development of CAMSA have undergone multiple rounds of expert review using the Delphi Method. The evaluation results of CAMSA utilize a combination of process evaluation and outcome evaluation, presenting the seven sequentially included sports skills and compiling them into a cohesive set of combinations. Specifically, CAMSA not only assesses the proficiency in each of the seven sports skills (skill score) but also quantifies the overall completion time for these skills (completion time) and establishes requirements for this completion time (completion score, or time score). This necessitates that participants perform each sports skill quickly and accurately during the CAMSA test, efficiently transition between different skills and movement directions, and minimize the timing score while maintaining the quality of their actions, thereby maximizing the overall test results (total score).

**FIGURE 1:**
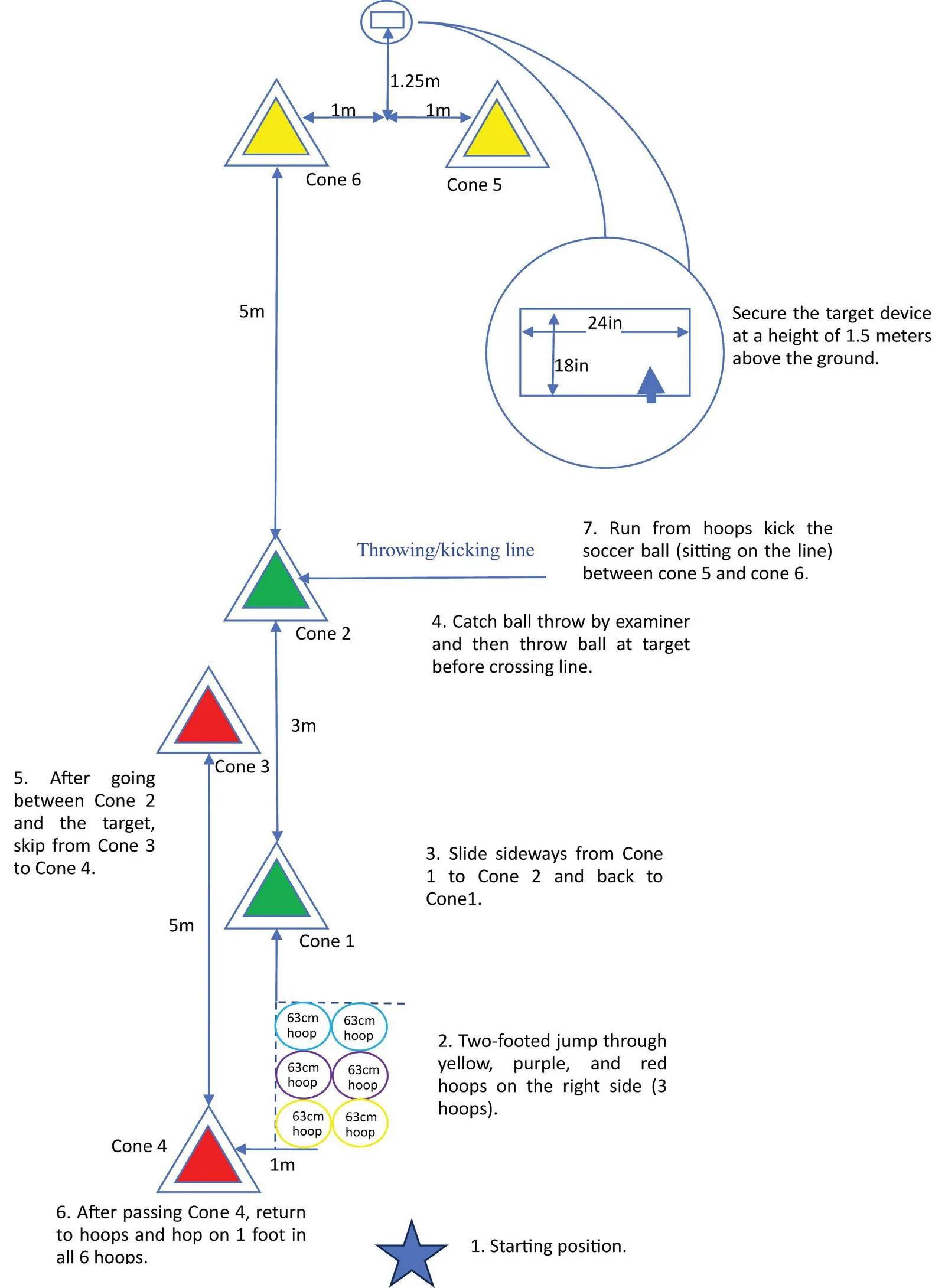
CAMSA test schematic diagram.

According to the CAMSA test requirements, participants are permitted to practice twice before the official test. During the first practice session, each action must be executed smoothly. Participants may be encouraged to slow down and focus on their movements. In the second practice session, the emphasis shifts to improving the quality of the actions while also increasing completion speed. During the formal test, participants are required to maximize their speed while maintaining the quality of their actions. Throughout both practice sessions and the formal test, testers provide verbal prompts but do not offer feedback on performance or attempt to encourage or modify the child’s execution. The timing starts when the “start” command is given and ends when the participant kicks the football shown in Figure 1. The whole test is recorded.

#### 2.2.2 Fundamental movement skills test

Test of Gross Motor Development-Third Edition (TGMD-3) was developed by Ulrich et al. and demonstrates strong reliability and validity for children aged 3–12 years in China (Li et al., 2022). This tool is designed to assess the developmental level of children’s fundamental movement skills. The test indicators of the TGMD-3 primarily include locomotor and ball skills. The TGMD-3 test includes 13 test indicators, including run, gallop, hop, skip, horizontal jump, slide, dribble, overhand throw, underhand throw, two hand strike, one-hand strike, catch, and kick. In this study, the same group of subjects was assessed using the TGMD-3 to verify the concurrent validity of the CAMSA skill component. A total of 150 students were recruited for the test; however, 8 students were excluded, resulting in a final sample size of 142 participants (mean age: 9.97 ± 1.39 years), including 66 boys and 76 girls. When stratified by single-year age groups, the sample distribution was as follows: 27 participants (11 boys, 16 girls) in the 8-year-old group, 31 participants (16 boys, 15 girls) in the 9-year-old group, 29 participants (13 boys, 16 girls) in the 10-year-old group, 29 participants (16 boys, 13 girls) in the 11-year-old group, and 26 participants (10 boys, 16 girls) in the 12-year-old group. The assessment was conducted by the standard testing procedures of TGMD-3 and was administered by one physical education teacher and two graduate students.

### 2.3 Data processing

All quantitative analyses were performed using IBM SPSS Statistics 26.0 for descriptive statistics, reliability estimation, and preliminary correlational analyses, while IBM AMOS 26.0 was employed for confirmatory factor analysis (CFA) to examine the hypothesized latent factor structure. Referencing established psychometric protocols for performance-based motor skill assessments in the sports science literature (Menescardi et al., 2022), this study implemented a standardized, multi-faceted framework to systematically evaluate the reliability and validity of the adapted Chinese version of the CAMSA. For validity evaluation, three complementary domains were examined: structural validity, content validity, and concurrent validity.

## 3 Results

### 3.1 Children’s CAMSA levels are significantly positively correlated with age

The results indicated that as age increased, the scores for fundamental movement skills among the five age groups of 8–12 years exhibited an upward trend (see Table 1) and were positively correlated with age (r time score = 0.414, r skill score = 0.305, r total score = 0.456, *P* < 0.01).

**TABLE 1:** Mean scores of children’s CAMSA at different ages (*N* = 1,160).

Age	Time Score	Skill Score	Total Score
8	5.47 ± 2.48	9.00 ± 2.00	14.47 ± 3.53
9	6.93 ± 2.71	9.65 ± 1.88	16.58 ± 3.75
10	7.21 ± 2.72	10.20 ± 1.79	17.41 ± 3.41
11	8.10 ± 2.43	10.09 ± 1.88	18.19 ± 3.35
12	9.02 ± 2.41	10.81 ± 1.36	19.83 ± 2.82
Age-related	0.414**	0.305**	0.456**

**P < 0.01.

### 3.2 Item difficulty, discrimination, and concurrent validity

#### 3.2.1 Item difficulty

The difficulty of an item refers to the challenge posed by the test content to the test subject and is primarily calculated using the formula: item difficulty = average score/full score. The overall difficulty of the test should be moderate. If the difficulty is excessively high, the test subjects’ scores tend to be low, resulting in a negatively skewed distribution. Conversely, if the difficulty is too low, the test subjects’ scores are generally high, leading to a positively skewed distribution. Both excessively high and low difficulty levels can diminish the variability in test scores among test takers, thereby reducing the reliability and discriminative power of the assessment.

Item difficulty indices were evaluated for the CAMSA total score, skill score and time score. The mean difficulty value was 0.62 for the total score, 0.71 for the skill score, and 0.52 for the time score, with corresponding difficulty ranges of 0.52–0.71, 0.64–0.77 and 0.39–0.64 respectively (see Table 2). According to established criteria, difficulty values between 15% and 85% are regarded as acceptable (Ahastasi and Urbina, 1997). Overall, all three sets of difficulty indices fall within the reasonable range and meet standard psychometric requirements. Consistent with international evidence (Webster and Ulrich, 2017), CAMSA total score, time score and skill score all show a progressive increase with age, which aligns with the fundamental developmental principle that children’s fundamental movement skills improve as they grow older.

**TABLE 2:** CAMSA test difficulty coefficients for different age groups (*N* = 1,160).

Age	Time Score	Skill Score	Total score
8	0.39	0.64	0.52
9	0.46	0.69	0.59
10	0.52	0.73	0.61
11	0.57	0.72	0.65
12	0.64	0.77	0.71
Overall	0.52	0.71	0.62

#### 3.2.2 Item discrimination

Item discrimination, often referred to simply as discrimination, denotes the ability of a test item to differentiate between subjects of varying proficiency levels. It reflects the extent to which the test aligns with the actual performance level of the subjects. If a high score on a specific task indicates strong mastery of that task, while a low score suggests poor mastery, then the test demonstrates effective discrimination. Typically, item discrimination is represented by the letter D. The calculation involves taking the average score of the top 27% of performers, subtracting the average score of the bottom 27%, and then dividing the result by the maximum possible score.

The study revealed that the average score of the high-scoring group in the CAMSA was 21.98, while the average score of the low-scoring group was 12.22, resulting in a discrimination value of 0.35. Additionally, the average score of the high-scoring group in the CAMSA skill score was 12.16, compared to an average score of 7.43 for the low-scoring group, yielding a discrimination value of 0.34. Furthermore, the average score of the high-scoring group in the CAMSA time score was 11.02, whereas the low-scoring group had an average score of 3.77, which produced a discrimination value of 0.52. The discrimination values for the CAMSA total score, skill score, and time score were 0.35, 0.34, and 0.52, respectively, all exceeding the threshold of 0.30. Related studies indicate that items with a discrimination value greater than 0.30 are considered acceptable (Liu and Chen, 1997). These results demonstrate that the CAMSA total score, skill score, and time score exhibit strong item discrimination.

### 3.3 Concurrent validity

Concurrent validity refers to the degree of consistency between a selected test method and a known benchmark. It is a type of criterion-related validity, also referred to as concurrent validity or compatible validity. This study utilized the TGMD-3 as the gold standard, excluding data that were missing, untested, or from subjects who were sick or unwell. A total of 150 subjects were included to explore the correlation between the total score of the CAMSA (17.82 ± 3.80) and the total score of the TGMD-3 (56.57 ± 22.97) (*R* = 0.613, *P* < 0.001). The findings indicated a moderately significant correlation between CAMSA and TGMD-3, suggesting that CAMSA can serve as a valid tool for assessing children’s fundamental movement skills.

### 3.4 Item reliability

This research conducted a 2-week follow-up assessment with 182 participants who had completed the initial test. After excluding 7 participants with incomplete data, 175 valid participants were included in the final analysis, with a mean age of 9.98 years (SD = ± 1.13). This study employed the two-way mixed-effects, absolute-agreement ICC(3,1) model to assess inter-rater reliability and test-retest reliability for the adapted Chinese CAMSA: the time performance component, the skill mastery component, and the overall composite scale. All reliability estimates were reported with 95% bootstrap confidence intervals, in strict accordance with established psychometric standards for pediatric motor competence assessments.

#### 3.4.1 Test-retest reliability

For test-retest reliability, it is widely accepted that a correlation coefficient below 0.40 indicates low correlation, 0.40–0.70 indicates moderate correlation, and above 0.70 indicates high correlation (Akoglu, 2018). As shown in Table 3, the Pearson correlation coefficients between the two rounds of assessments were 0.814 for skill scores, 0.777 for time scores, and 0.850 for total scores (all *P* < 0.01). All 7 individual motor skill items and 3 composite scores in the table achieved highly significant positive correlations, with every correlation coefficient exceeding the 0.70 threshold for acceptable test-retest reliability in children’s motor competence assessment. The narrow 95% confidence intervals for composite scores (total score 95% CI [0.805, 0.888], skill score 95% CI [0.743, 0.872]) further confirm that the high stability of the adapted Chinese CAMSA is not a random sampling artifact. The Pearson correlation analysis was strictly performed on the approximately normally distributed continuous scores, fully satisfying the normality and linearity assumptions of the statistical method. This set of results collectively demonstrates that the adapted Chinese version of CAMSA has excellent cross-time measurement stability, providing a solid psychometric foundation for its application in children’s motor skill screening. This finding is consistent with the favorable test-retest reliability results reported by Cristina, Menescardi et al. in their Spanish study (Menescardi et al., 2022).

**TABLE 3:** Pearson correlation coefficient for the retest (*N* = 175).

Test indicators	First test	Second test	Correlation coefficient	Cronbach’s α	95% confidence intervals
2-foot Jumping	1.97 ± 0.18	1.96 ± 0.20	0.763**	0.864	[0.432, 1.000]
Sliding	2.17 ± 0.82	2.10 ± 0.86	0.874**	0.932	[0.814, 0.921]
Catching	0.71 ± 0.46	0.73 ± 0.45	0.761**	0.864	[0.649, 0.864]
Throwing	0.43 ± 0.58	0.41 ± 0.60	0.704**	0.826	[0.576, 0.824]
Skipping	0.71 ± 0.65	0.67 ± 0.60	0.726**	0.839	[0.600, 0.842]
One-foot hopping	1.54 ± 0.60	1.50 ± 0.61	0.747**	0.855	[0.613, 0.850]
Kicking	0.83 ± 0.66	0.79 ± 0.65	0.798**	0.888	[0.704, 0.878]
Time score	6.85 ± 2.87	7.43 ± 2.80	0.777**	0.874	[0.711, 0.834]
Skill score	8.36 ± 1.79	8.17 ± 1.84	0.814**	0.897	[0.743, 0.872]
Total raw score	15.21 ± 3.86	15.61 ± 3.85	0.850**	0.919	[0.805, 0.888]

**P < 0.01.

#### 3.4.2 Rater reliability

Rater reliability refers to the degree of consistency in test results produced by two or more evaluators when administering the same assessment to an identical subject. A high level of reliability is indicated by consistent results across different raters, while substantial discrepancies among raters raise concerns regarding the reliability of the measurement instrument. This reliability is commonly quantified through the rater correlation coefficient. In the current study, after undergoing standardized training, two raters simultaneously assessed 139 randomly selected students (mean age 9.43 ± 0.85 years). The scores assigned by the two evaluators were subsequently analyzed using the Spearman correlation coefficient. The objectivity and accuracy of the raters’ evaluations of the test items play a crucial role in determining the overall rater reliability (Sun and Zhang, 2005).

The research results revealed that the correlation coefficients between the scores assigned by the two evaluators in the CAMSA skill section were largely above 0.70, with the exception of the hopping. The overall skill section yields a correlation coefficient of 0.626, demonstrating a statistically significant association. For the categorical scoring dimension, Cohen’s Kappa values, which correct for chance agreement, varied from 0.679 to 0.919. Five out of seven individual skills achieved Kappa values above 0.80, representing an almost perfect level of inter-rater agreement. Only the sliding (κ = 0.683) and one-foot hopping (κ = 0.679) items fell into the substantial agreement range, a pattern consistent with the inherent variability of young children’s dynamic movement performance during in-person assessment (details are presented in Table 4).

**TABLE 4:** Pearson correlation coefficients for the skill scores assigned by various evaluators (*N* = 139).

Test index	Scorer A	Scorer B	Correlation coefficient	Kappa	Cronbach’s α	95% confidence intervals
2-foot Jumping	1.96 ± 0.20	1.95 ± 0.22	0.922**	0.919	0.958	[0.705, 1.000]
Sliding	2.09 ± 0.86	2.19 ± 0.90	0.835**	0.683	0.908	[0.762, 0.900]
Catching	0.71 ± 0.45	0.76 ± 0.43	0.821**	0.816	0.901	[0.710, 0.920]
Throwing	0.46 ± 0.57	0.47 ± 0.58	0.835**	0.846	0.896	[0.662, 0.925]
Skipping	0.67 ± 0.70	0.68 ± 0.68	0.873**	0.880	0.935	[0.776, 0.952]
One-foot hopping	1.57 ± 0.59	1.52 ± 0.56	0.689**	0.679	0.807	[0.514, 0.806]
Kicking	0.92 ± 0.65	0.92 ± 0.62	0.839**	0.819	0.916	[0.733, 0.923]
Skill score	8.37 ± 1.96	8.56 ± 1.97	0.626**		0.782	[0.525, 0.743]

**P < 0.01.

Internal consistency, measured by Cronbach’s α, was satisfactory across all indicators. All individual skill items obtained α values above 0.80, with the two-foot jumping item reaching 0.958. The total composite score yielded a Cronbach’s α of 0.782, exceeding the conventional 0.70 threshold for acceptable research instruments. The 95% confidence intervals for all reliability estimates did not cross zero, with lower bounds ranging from 0.514 to 0.776, further confirming the statistical robustness of the observed reliability results (details are presented in Table 4). These findings imply that the reliability of the scorers for the CAMSA skill section is commendable.

In the context of rater reliability, the CAMSA scale utilizes a dual methodology that incorporates both process evaluation and outcome evaluation, with a particular emphasis on skill and time variables. Given the consistency observed in the results pertaining to the time variable, the analysis of rater reliability was limited to the skill variable of CAMSA. Prior to conducting the evaluations, both raters received training and subsequently assessed a sample of 139 students, following established scoring criteria. The inter-rater reliability analysis for the CAMSA skill component revealed a moderate level of agreement across independent raters, with a statistically significant Spearman correlation coefficient of 0.642 (*p* < 0.001). Concurrently, item-level Cohen’s kappa coefficients demonstrated substantial agreement for all assessed items.

### 3.5 Construct validity and criterion validity

To rigorously avoid the well-documented overfitting risk that arises when exploratory and confirmatory analyses are conducted on the same full dataset, the total sample of 1,160 participants in this study was randomly split into two demographically matched independent subsamples of equal size (*n* = 580 each), strictly following the COSMIN psychometric standards for instrument validation. The first subsample was exclusively allocated for exploratory analysis, where we first verified the dataset suitability for dimension reduction: the KMO value reached 0.599, and Bartlett’s sphericity test showed highly significant results (χ^2^ = 267.89, df = 21, *p* < 0.001), confirming sufficient inter-item correlations to support subsequent factor extraction.

Given all 7 CAMSA items are scored on an ordinal 0–3 rating scale, we adopted Categorical Principal Components Analysis (CATPCA)—a method purpose-built for ordinal categorical variables—to process the data. Unlike conventional exploratory factor analysis that treats ordinal scores as continuous data, CATPCA preserves the original ordinal attributes of the skill ratings through optimal scaling transformation. Following the eigenvalue-greater-than-1 criterion, two theoretically interpretable dimensions were extracted, with all item communalities ranging from 0.305 to 0.602, and the two factors together accounting for 69.1% of the total variance. After 3 iterations of Varimax orthogonal rotation, a clear simple structure was formed: Factor 1 (object control skills) loaded with kick, catch and throw (standardized loadings 0.609–0.775), Factor 2 (locomotor skills) loaded with slide, skip, jump and hop (standardized loadings 0.546–0.705), with nearly zero cross-loadings between the two factors. The full rotated component matrix is presented in Table 5.

**TABLE 5:** Two-factor structure of the revised CAMSA: rotated component loadings.

Index component	Common	
	Factor 1	Factor 2
Kicking	0.609	0.634
Catching	0.757	
Throwing	0.775	
Sliding		
Skipping		0.705
2-foot jumping		0.546
One-foot hoping		0.555

The cumulative variance explained by the 2 extracted dimensions reached 69.62%, which satisfies the generally accepted 60% threshold for exploratory factor analysis in behavioral research. The overall Cronbach’s alpha of the 2- dimensional model is 0.892, indicating good internal consistency of the extracted factor structure. Previous studies have indicated that the Canadian Agility and Movement Skill Assessment test (CAMSA) effectively evaluates children’s fundamental movement skills, specifically in the domains of mobility and manipulation.

Following the two-factor structure preliminarily extracted via varimax-rotated Categorical Principal Components Analysis (CATPCA) in the exploratory phase, we proceeded to conduct confirmatory factor analysis (CFA) on a fully independent split subsample to rigorously test the model-data fit of this hypothesized two-dimensional measurement framework. Notably, this second subsample shared zero overlapping participants with the dataset used for exploratory structure extraction, eliminating the risk of circular overfitting. This independent holdout sample was exclusively reserved for the subsequent CFA procedure, to formally validate the empirical stability and goodness-of-fit of this pre-specified two-dimensional structure, thereby generating unbiased, methodologically rigorous evidence for the structural validity of the culturally adapted Chinese version of the CAMSA.

According to relevant research, confirmatory factor analysis emphasizes the importance of several fit indices in evaluating alternative models, particularly the Comparative Fit Index (CFI), Normed Fit Index (NFI), and Root Mean Square Error of Approximation (RMSEA) (Wu, 2013). Specifically, a CFI and NFI value exceeding 0.90 indicates an acceptable fit for the hypothesized model, while values greater than 0.95 suggest an excellent fit. Regarding the RMSEA, a value below 0.08 indicates an acceptable model fit, and a value below 0.05 signifies a very good fit.

**FIGURE 2:**
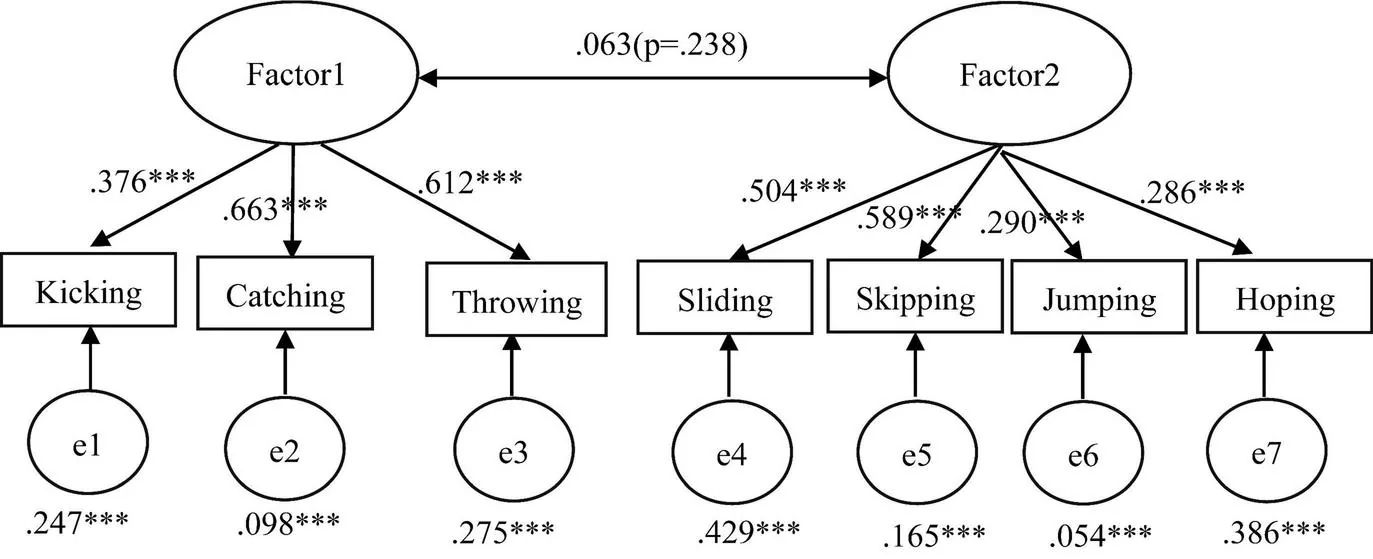
Confirmatory factor analysis. ***p < 0.001. The path coefficients listed in the figure are standardized coefficients.

In the context of structural validity, the exploratory factor analysis of the skills component revealed the presence of two distinct common factors. The first common factor comprised three indicators: catching the ball, throwing the ball with an overhand motion, and kicking a stationary ball, all of which pertain to object control skills. The second common factor included four indicators: hopping on one foot, sliding steps, jumping steps, and again, hopping on one foot, which are categorized as locomotor skills. These findings indicate that the CAMSA effectively assesses fundamental movement skills in children. Furthermore, the confirmatory factor analysis of the two-factor model yielded the following fit indices: χ^2^/df = 3.849, CFI = 0.931, GFI = 0.988, TLI = 0.889, AGFI = 0.975, NFI = 0.911, RMR = 0.012, RMSEA = 0.050, AIC = 80.033, BIC = 155.875. Collectively, these results suggest that CAMSA demonstrates strong structural validity, with the object control skills and locomotor skills yielding acceptable internal consistency estimates of McDonald’s ω≈0.606 and CR≈0.606, and ω≈0.501 and CR≈0.501, respectively, which are consistent with the expected reliability level for a children’s gross motor competence assessment tool (details are presented in Figure 2).

In stark contrast, the unidimensional one-factor model, which constrained all seven items to load directly onto a single general motor competence latent variable, demonstrated severely inadequate fit: χ^2^/df = 16.675, CFI = 0.593, TLI = 0.390, RMSEA = 0.116, SRMR = 0.029. The nested model comparison further confirmed that the two-factor correlated model was statistically superior to the unidimensional alternative [Δχ^2^(1) = 183.413, *p* < 0.001], with substantially lower values on both information criteria (AIC = 80.033, BIC = 155.875). These results collectively indicate that the correlated two-factor structure, which delineates object control and locomotor skills as two distinct subcomponents under the overarching construct of general motor competence, provides a far more accurate and theoretically consistent representation of the CAMSA’s underlying measurement structure than the unidimensional specification.

Currently, there exist two primary methodologies for evaluating students’ FMS. The first approach involves administering a variety of tasks and interpreting the resulting scores based on age classifications. In this context, the evaluation procedures become increasingly intricate for older children, as exemplified by the Movement Assessment Battery for Children (MABC). The second approach employs a uniform set of tasks across all age groups, while adjusting the performance standards or scoring criteria to account for anticipated age-related differences in performance, as seen in the Test of Gross Motor Development (TGMD). The present study utilized an evaluation method akin to TGMD, particularly concerning its criterion validity. Furthermore, TGMD is recognized and widely implemented in various countries, lending it considerable authority. Criterion-related validity analysis revealed a strong, statistically significant positive association between the CAMSA total score and the TGMD-3 total score, with a Pearson correlation coefficient of *r* = 0.613(*P* = 0.000). Beyond the correlation estimate, the corresponding coefficient of determination R^2^ was reported as 0.376, demonstrating that the two well-validated FMS assessment instruments share 37.6% of their explained variance.

### 3.6 Shanghai Norms

Initially, we employed age and gender as independent variables, while utilizing time score, skill score, and total score as dependent variables to perform a variance analysis aimed at identifying potential differences among children of varying ages and genders. The normative sample was derived from two schools located in urban areas, two schools situated in suburban regions, and one school positioned on the urban-rural fringe. The student distribution was recorded as 412:396:352, with a gender ratio of boys to girls being 1:1.04.

As shown in Table 6, a 2 (gender) × 5 (age: 8–12 years) two-way analysis of variance was conducted on three dependent variables, namely time score, skill score and total raw score. For the main effect of gender, statistical significance was only detected in skill score (*F* = 5.991, df = 1, *P* = 0.015, η*p*^2^ = 0.005). No significant gender difference was found in time score (*F* = 1.816, df = 1, *P* = 0.178, η*p*^2^ = 0.002) or total raw score (*F* = 0.094, df = 1, *P* = 0.759, η*p*^2^ < 0.001). For the main effect of age, highly significant differences were observed across all three indicators: time score (*F* = 70.884, df = 4, *P* < 0.001, η*p*^2^ = 0.198), skill score (*F* = 32.861, df = 4, *P* < 0.001, η*p*^2^ = 0.103) and total raw score (*F* = 85.683, df = 4, *P* < 0.001, η*p*^2^ = 0.230), indicating age as the primary source of performance variance in this sample. For the gender × age interaction effect, significant interactions were identified in time score (*F* = 28.029, df = 4, *P* < 0.001, η*p*^2^ = 0.089) and total raw score (*F* = 15.344, df = 4, *P* < 0.001, η*p*^2^ = 0.051), while the interaction on skill score did not reach statistical significance (*F* = 0.819, df = 4, *P* = 0.513, η*p*^2^ = 0.003). Since significant main or interaction effects exist in at least one core dimension across all adjacent age subgroups, merging non-significant groups would mask the inherent developmental heterogeneity of test performance. To ensure the validity and applicability of the norm system in physical fitness assessment practice, this study retains all 5 discrete age subgroups without aggregation, and will subsequently develop age-stratified grade norms and percentile norms for Shanghai children aged 8–12 years.

**TABLE 6:** Analysis of variance of gender and age in children’s CAMSA.

Variate	Items	SS	df	*F*	*P*	η *p*^2^
	Time score	10.917	1	1.816	0.178	0.002
Gender	Skill score	19.176	1	5.991	0.015	0.005
	Total raw score	1.030	1	.094	0.759	0.000
	Time score	426.013	4	70.884	0.000	0.198
Age	Skill score	105.185	4	32.861	0.000	0.103
	Total raw score	937.951	4	85.683	0.000	0.230
	Time score	168.456	4	28.029	0.000	0.089
Gender*Age	Skill score	2.620	4	.819	0.513	0.003
	Total raw score	167.965	4	15.344	0.000	0.051

SS, sum of squares.

Prior to the establishment of the standard score norm, it is essential to standardize and transform the sample data. The conversion to standard scores necessitates that the original data exhibit a normal distribution. Consequently, this study performed a normality test on the time part score, skill part score, and total score. If the sample data’s skewness and kurtosis values lie within ± 2, this suggests that the data follow a normal distribution and are appropriate for transformation into standard scores as shown in Table 7.

**TABLE 7:** CAMSA scores and normality test.

Variate	Gender	Age (year)	M ± SD	S_k_ ± SE	K ± SE
Total score	Male	8	15.106 ± 3.511	−0.037 ± 0.227	−0.189 ± 0.451
		9	16.901 ± 3.855	−0.295 ± 0.212	−0.226 ± 0.420
		10	17.853 ± 3.456	0.099 ± 0.231	0.206 ± 0.459
		11	18.431 ± 3.578	−0.385 ± 0.231	−0.117 ± 0.459
		12	18.448 ± 2.212	0.311 ± 0.217	0.076 ± 0.430
	Female	8	13.845 ± 3.453	−0.155 ± 0.225	−0.399 ± 0.446
		9	16.241 ± 3.625	−0.163 ± 0.217	−0.643 ± 0.431
		10	17.000 ± 3.338	−0.012 ± 0.227	−0.547 ± 0.451
		11	17.955 ± 3.096	−0.119 ± 0.230	−0.187 ± 0.457
		12	21.400 ± 2.624	0.171 ± 0.230	−0.516 ± 0.457
Time score	Male	8	5.858 ± 2.431	0.312 ± 0.227	−0.336 ± 0.451
		9	7.252 ± 2.794	−0.057 ± 0.212	−0.385 ± 0.420
		10	7.422 ± 2.719	0.163 ± 0.231	−0.516 ± 0.459
		11	8.303 ± 2.386	−0.038 ± 0.231	−0.454 ± 0.459
		12	7.512 ± 1.903	0.455 ± 0.217	0.843 ± 0.430
	Female	8	5.095 ± 2.485	0.281 ± 0.225	−0.410 ± 0.446
		9	6.596 ± 2.597	0.123 ± 0.217	−0.813 ± 0.431
		10	7.009 ± 2.717	0.412 ± 0.227	−0.675 ± 0.451
		11	7.909 ± 2.481	−0.034 ± 0.230	−0.551 ± 0.457
		12	10.727 ± 1.675	−0.309 ± 0.230	1.757 ± 0.457
Skill score	Male	8	9.248 ± 2.085	−0.355 ± 0.227	−0.211 ± 0.451
		9	0.649 ± 1.941	−0.216 ± 0.212	−0.258 ± 0.420
		10	10.431 ± 1.863	−0.511 ± 0.231	0.097 ± 0.459
		11	10.128 ± 2.169	−0.573 ± 0.231	−0.274 ± 0.459
		12	10.936 ± 1.262	−0.538 ± 0.217	−0.019 ± 0.430
	Female	8	8.750 ± 1.892	−0.345 ± 0.225	−0.396 ± 0.446
		9	9.645 ± 1.826	−0.778 ± 0.217	0.474 ± 0.431
		10	9.991 ± 1.693	−0.672 ± 0.227	0.230 ± 0.451
		11	10.046 ± 1.559	−0.240 ± 0.230	−0.358 ± 0.457
		12	10.673 ± 1.460	−0.006 ± 0.230	−0.917 ± 0.457

S_k_, skewness; SE, standard error; K, Kurtosis.

The findings indicated that the peak and skewness values for the 8–12 age group across different genders fell within the range of ± 2. This suggests that the original data pertaining to the time part scores, skill part scores, and total scores for both boys and girls within the 8–12 age bracket adhered to a normal distribution (refer to Table 7). Consequently, this study transformed the original scores into standard Z scores and subsequently categorized these scores into grade norms ranging from 1 to 5, based on the established Z score groupings of < −2, −2 to −1, −1 to 1, 1 to 2, and > 2 (see Table 8) and percentile norms (see Table 9).

**TABLE 8:** CAMSA (revised version) developmental level norms for children aged 8–12 in Shanghai (*N* = 1,160).

Age	Gender	Score	Rank				
			1	2	3	4	5
8	Male	Total score	1∼8	9∼11	12∼18	19∼22	23∼28
		Time score	–	1∼3	4∼8	9∼10	11∼14
		Skill score	1∼5	6∼7	8∼11	12∼13	14
	Female	Total score	1∼6	7∼10	11∼17	18∼20	21∼28
		Time score	–	1∼2	3∼7	8∼10	11∼14
		Skill score	1∼4	5∼6	7∼10	11∼12	13∼14
9	Male	Total score	1∼9	10∼13	14∼20	21∼24	25∼28
		Time score	1	2∼4	5∼10	11∼12	13∼14
		Skill score	1∼5	6∼7	8∼11	12∼13	14
	Female	Total score	1∼8	9∼12	13∼19	20∼23	24∼28
		Time score	1	2∼3	4∼9	10∼11	12∼14
		Skill score	1∼5	6∼7	8∼11	12∼13	14
10	Male	Total score	1∼10	11∼14	15∼21	22∼24	25∼28
		Time score	1	2∼4	5∼10	11∼12	13∼14
		Skill score	1∼6	7∼8	9∼12	13∼14	–
	Female	Total score	1∼10	11∼13	14∼20	21∼23	24∼28
		Time score	1	2∼4	5∼9	10∼12	13∼14
		Skill score	1∼6	7∼8	9∼11	12∼13	14
11	Male	Total score	1∼11	12∼14	15∼22	23∼25	26∼28
		Time score	1∼3	4∼5	6∼10	11∼13	14
		Skill score	1∼5	6∼7	8∼12	13∼14	–
	Female	Total score	1∼11	12∼14	15∼21	22∼24	25∼28
		Time score	1∼2	3∼5	6∼10	11∼12	13∼14
		Skill score	1∼6	7∼8	9∼11	12∼13	14
12	Male	Total score	1∼14	15∼16	17∼20	21∼22	23∼28
		Time score	1∼3	4∼5	6∼9	10∼11	12∼14
		Skill score	1∼8	9	10∼12	13	14
	Female	Total score	1∼16	17∼18	19∼24	25∼26	27∼28
		Time score	1∼7	8∼9	10∼12	13∼14	–
		Skill score	1∼7	8∼9	10∼12	13	14

**TABLE 9:** Percentile norms of CAMSA (revised version) scores for children aged 8–12 years in Shanghai (*N* = 1,160).

Content	p-th percentile	8 year		9 year		10 year		11 year		12 year	
		Male	Female	Male	Female	Male	Female	Male	Female	Male	Female
Total score	5	8	7	8	9	12	11	11	12	14	15
	10	10	9	10	10	12	12	12	13	15	17
	25	13	12	15	14	15	14	16	16	17	20
	50	15	14	17	16	18	17	18	18	19	21
	70	17	16	20	19	20	20	21	20	20	23
	90	19	17	21	20	21	20	22	21	21	24
	95	21	19	22	21	23	22	23	22	22	25
Time score	5	1	1	2	2	2	1	4	3	4	6
	10	2	1	3	2	3	3	4	4	4	6
	25	4	3	5	5	5	5	7	6	6	9
	50	6	5	7	6	7	7	8	8	8	11
	75	8	7	9	8	9	9	9	10	8	12
	90	8	7	10	9	10	10	10	10	10	12
	95	10	9	10	10	11	11	11	11	10	13
Skill score	5	5	4	5	5	6	7	5	6	8	8
	10	5	5	6	6	7	7	6	7	8	8
	25	8	7	9	9	9	9	9	9	10	9
	50	9	9	10	10	11	10	10	10	11	11
	75	11	10	11	11	12	11	12	11	12	12
	90	11	10	11	10	12	11	12	11	12	12
	95	12	12	12	12	13	12	13	12	13	13

## 4 Discussion

### 4.1 The development of CAMSA levels in accordance with the principles of children’s motor development

As children mature, there is a consistent increase in CAMSA scores, which demonstrates a positive correlation with age. This finding indicates that CAMSA aligns with the established principles of children’s motor development and exhibits a measurable degree of advancement. As children age, their physical and cognitive capabilities, as well as their experiences in motor practice, become increasingly enriched. Consequently, the CAMSA assessment can be regarded as an effective tool for evaluating the motor skill levels of students across various age groups. In the Chinese educational context, K-12 students receive standardized, nationally aligned physical education curricula with clearly defined grade-specific teaching objectives and structured learning content. As students advance through successive school grades, their systematic exposure to specialized motor skill instruction progressively deepens, resulting in significantly higher mastery of foundational and advanced movement skills among older cohorts relative to their younger peers. Similar to previous assessments (Dania et al., 2020; Kaioglou et al., 2020), the CAMSA score was found to be related to the gender and age of participants, with boys performing better than girls and scores increasing with age.

### 4.2 Appropriateness of CAMSA test item difficulty and discrimination for children in Shanghai

The evaluation of item difficulty and discrimination coefficients for the CAMSA test demonstrates that these metrics fall within acceptable parameters and exhibit satisfactory values. The study findings indicate a progressive decline in difficulty coefficients for both CAMSA subtests and the overall assessment as children age, suggesting that the test items become increasingly less challenging and more accessible with maturation. This pattern reflects a reduction in the developmental demands imposed by the CAMSA test over time, corresponding to improvements in children’s agility and motor skills. Additionally, the results reveal that the challenges associated with skill acquisition exceed those related to time constraints, consistent with the developmental characteristics of children and established principles of motor skill cognition. Given that motor skill development requires the coordinated activation of multiple joints, skill acquisition in young children is marked by substantial physical collaboration and coordination, necessitating the simultaneous engagement of multiple limbs during imitation and task execution.

### 4.3 Reliability and validity of the CAMSA test among children in Shanghai

The results of the present study provide mixed but overall supportive evidence for the properties of the CAMSA test in children aged 8–12 years in Shanghai. While the test demonstrated adequate internal consistency for research use and its overall model fit was satisfactory, we also note that several indicators (specifically Jumping, Hopping, and Kicking) exhibited relatively low standardized factor loadings and modest composite reliability estimates. These findings suggest that the CAMSA is suitable for evaluating general agility and motor skill development in this population, yet caution is warranted when interpreting scores from these specific, weakly loaded items.

The observed reliability and validity indices were largely comparable to those reported in prior international studies (Lander et al., 2017; Longmuir et al., 2017; Menescardi et al., 2022), which lends preliminary support to the cross-cultural applicability of the test. Importantly, the proposed two-factor structure demonstrated significantly better fit than a unidimensional model, supporting its incremental validity over a general motor competence construct.

The identified low factor loadings for specific motor tasks (e.g., hopping, jumping) imply that these items do not robustly reflect their hypothesized domain-specific latent factors. This attenuation may stem from multiple sources: the scoring system’s potential inability to fully capture the coordinative complexity of such tasks, the pronounced developmental heterogeneity within the childhood sample, and contextual factors in Shanghai that might lead to differential skill acquisition.

An important observation from the pattern of factor loadings in the present study suggests that these items exhibit substantial associations with a general motor competence factor, a trend that could be formally tested in future research using a bifactor model. This supports an interpretation where the items function more as indicators of global motor proficiency. In light of this, and in line with a balanced interpretation of the psychometric properties, we note that the composite reliability estimates for the domains (CR and ω≈ 0.50–0.61) are modest, falling below the conventional 0.70 threshold. This underscores the challenge of achieving high internal consistency with a limited number of complex performance-based indicators, and cautions against over-interpreting the domain-specificity of weakly loading items. Future investigations could aim to refine the measurement of these tasks, or formally evaluate a bifactor structure to explicitly integrate them into an aggregate measure of general motor competence.

The evaluation of inter-rater reliability highlighted the critical role and effectiveness of rater training, providing robust evidence for the validity of the CAMSA scoring system. This study found strong agreement among raters. Furthermore, the CAMSA scores showed a significant positive correlation with those from the TGMD-3 assessment. Both instruments employ identical tasks across all age groups, although they differ in performance criteria or scoring methods to accommodate expected age-related variations. In contrast, the MABC assessment utilizes different tasks and interprets scores according to age categories, incorporating progressively complex procedures for older children.

Empirical evidence highlights the existence of gender differences in motor skill performance, with male children demonstrating superior raw scores relative to their female counterparts. A granular examination of individual skill components indicates that these disparities are primarily attributable to boys’ enhanced abilities in ball-related tasks, including catching, overhand throwing, and kicking stationary balls. These results corroborate prior research findings, which report that boys tend to outperform girls in activities such as kicking, overhand throwing, and catching, whereas girls show greater proficiency in jumping and fine motor coordination (Barnett et al., 2008; Lubans et al., 2010). Additionally, primary school students of different genders may exhibit divergent preferences for particular sports activities. It is also important to note that such gender differences may become increasingly pronounced in older children, particularly those aged over 12 years.

In summary, the Children’s Agility and Motor Skills Assessment (CAMSA) has demonstrated robust reliability and validity for assessing agility and motor skills among children in Shanghai, thereby confirming the instrument’s efficacy for this population.

### 4.4 Development of CAMSA normative standards for child assessment in Shanghai

This study presents the establishment of normative standards for the Canadian Agility and Movement Skill Assessment (CAMSA) tailored to children in Shanghai, employing a five-point standardized scoring system. Within this system, a score of 3 denotes the mean performance level, scores of 2 and 4 reflect moderate deviations from the mean, and scores of 1 and 5 correspond to extreme deviations. The adoption of these norms enables domestic sports professionals to conduct objective evaluations of fundamental movement skills among students. The results indicate the presence of gender differences in the skill assessment component; however, no statistically significant gender differences were detected in either the timing component or the overall CAMSA score. These observed variations in motor skill performance are likely attributable to developmental factors associated with psychological and physiological maturation processes.

Furthermore, the assessment of students’ physical development and athletic capabilities is influenced by a complex interplay of factors. Primarily, individual educators may adopt diverse pedagogical approaches in the design and implementation of instructional content aimed at fostering students’ physical growth and engagement in sports activities. Moreover, some educational institutions provide extracurricular club activities that are gender-specific, potentially introducing biases in the development of sports skills between male and female students. Although the Canadian Agility and Movement Skill Assessment (CAMSA) closely replicates real-world sports scenarios, it does not comprehensively evaluate a full spectrum of agile motor skills, such as bilateral coordination, rotational movements, dynamic balance, and climbing. This limitation necessitates caution when interpreting gender-based differences derived from CAMSA outcomes. Consequently, the revision and establishment of normative data that reflect the distinctive characteristics of children in Shanghai would enhance the accuracy of assessments pertaining to students’ agility and athletic proficiency. This study advocates for researchers and primary school physical education instructors to consider the adoption of CAMSA as an alternative to traditional assessment methodologies.

## 5 Conclusion

The CAMSA scale exhibits an appropriate level of difficulty and demonstrates strong item discrimination. It also shows satisfactory test-retest reliability, inter-rater reliability, criterion validity, and construct validity. Consequently, the scale is a reliable and valid tool for assessing fundamental movement skills in children aged 8–12 years. The results reveal no significant gender differences in completion time or overall scores; however, notable gender differences were observed in specific motor skill performance. This study has established normative data for grades 1 through 5, including percentile ranks, which can serve as a valuable reference for evaluating the development of basic motor skills in children.

One limitation of this study is that the current normative dataset was exclusively derived from samples in Shanghai, which limits the generalizability of the established percentiles to broader Chinese child populations. In future research, we will expand the sampling framework to cover multiple provinces and cities across China, so as to develop nationally representative norms for this fundamental movement skill assessment tool. Beyond the development of national norms, future research should systematically evaluate measurement invariance across sex and chronological age groups. This step will rigorously verify the structural consistency of the assessment scale across distinct demographic subgroups, ultimately enabling a more robust and unbiased evaluation of fundamental movement skills in Chinese children.

## Data Availability

The datasets presented in this article are not readily available because the data that support the findings of this study are available from the corresponding author, upon reasonable request. Requests to access the datasets should be directed to Haitan Wu: wuhaitan@shnu.edu.cn.
